# Childhood Lead Exposure in the Palestinian Authority, Israel, and Jordan: Results from the Middle Eastern Regional Cooperation Project, 1996–2000

**DOI:** 10.1289/ehp.8339

**Published:** 2006-01-24

**Authors:** Jamal Safi, Alf Fischbein, Sameer El Haj, Ramzi Sansour, Madi Jaghabir, Mohammed Abu Hashish, Hassan Suleiman, Nimer Safi, Abed Abu-Hamda, Joyce K. Witt, Efim Platkov, Steven Reingold, Amber Alayyan, Tamar Berman, Matti Bercovitch, Yogesh Choudhri, Elihu D. Richter

**Affiliations:** 1 Environmental Protection and Research Institute, Gaza, Palestinian Authority; 2 Sanz Medical Center, Netanya, and Selikoff Center for Environmental Health and Human Development, Ra'anana, Israel; 3 Center for Environmental and Occupational Health Sciences, Birzeit University, Birzeit, Palestinian Authority; 4 Department of Community and Preventive Medicine, School of Medicine, Jordan University, Amman, Jordan; 5 Kupat Holim Meuhedet, Yerka, Israel; 6 Hebrew University-Hadassah School of Public Health and Community Medicine, Jerusalem, Israel; 7 Department of Pediatrics, Maimonides Hospital, Brooklyn, New York, USA; 8 School of Medicine, New York University, New York, New York, USA; 9 Pharmacology and Clinical Toxicology Unit, Assaf Harofeh Medical Center, Zerifin, Israel; 10 Health Canada, Ottawa, Ontario, Canada

**Keywords:** ambient lead pollution, blood lead, childhood lead exposures, Middle East regional project

## Abstract

In the Middle East, the major sources of lead exposure have been leaded gasoline, lead-contaminated flour from traditional stone mills, focal exposures from small battery plants and smelters, and kohl (blue color) in cosmetics. In 1998–2000, we measured blood lead (PbB) levels in children 2–6 years of age in Israel, Jordan, and the Palestinian Authority (*n* = 1478), using a fingerstick method. Mean (peak; percentage > 10 μg/dL) PbB levels in Israel (*n* = 317), the West Bank (*n* = 344), Jordan (*n* = 382), and Gaza (*n* = 435) were 3.2 μg/dL (18.2; 2.2%), 4.2 μg/dL (25.7; 5.2%), 3.2 μg/dL (39.3; < 1%), and 8.6 μg/dL (> 80.0; 17.2%), respectively. High levels in Gaza were all among children living near a battery factory. The findings, taken together with data on time trends in lead emissions and in PbB in children in previous years, indicate the benefits from phasing out of leaded gasoline but state the case for further reductions and investigation of hot spots. The project demonstrated the benefits of regional cooperation in planning and carrying out a jointly designed project.

The detection and prevention of lead toxicity and poisoning among children have been a major international public health priority. The distribution and severity of lead toxicity are determined largely by lead in gasoline emissions, proximity to environmental sources, point sources, hot spots, and episodic exposures, sometimes from food sources (Richter 1992). Infants and children are more vulnerable to lead exposure because of more rapid airway and gastrointestinal absorption, hand-to-mouth activities, and increased susceptibility of the developing brain to the neurotoxic effects of lead (Grigg 2004; Needleman 2004). High exposures produce acute poisoning, with abdominal pain, constipation, anemia, irritability, bone and joint pain, and convulsions. Lead exposure has been linked to increased risk for diverse health outcomes, including infertility and cancer (Lustberg and Silbergeld 2002; Silbergeld 2003).

Today, the major concerns regarding childhood lead exposures are the health risks associated with exposures once considered “normal.” These low-level exposures produce subtle and not readily detectable neurobehavioral effects on personality, intellectual development, behavior, and achievement (Delville 1999; Mendelsohn et al. 1998). New data suggest that a safe threshold without adverse neurobehavioral effect is lower than the U.S. Centers for Disease Control and Prevention threshold of 10 μg/dL for intervention, and that in fact a safe level may not be detectable (Canfield et al. 2003; Chiodo et al. 2004).

In the Middle East today, the major reported point sources of lead exposure are industrial sources, including smelters, battery factories, and radiator repair shops; flour from traditional stone mills; and the occasional burning of wastes (El Sharif et al. 2000; Hershko et al. 1989). Leaded gasoline remains the major source of environmental lead pollution, and > 300,000 tons of leaded gasoline are sold annually in Israel and the Palestinian Authority (Israel Ministry of Infrastructure 2004).

Children of lead-exposed workers are at high risk for lead poisoning (Friedman et al. 2005). Exposure and elevated blood lead (PbB) levels in children also comes from the use of traditional cosmetic kohl (*kahal*) among women and young girls (Nir 1992; Parry 1991). Isolated cases of lead poisoning from the use of dental powders such as Saoott and Cebagin, which can contain as much as 51% lead, have been reported (Abdullah 1984).

Information has not been readily available on “background” community-wide sources, notably from gasoline and general air pollution by lead and from high-risk spots in crowded areas. Past work in Israel suggested nontrivial risks from lead exposure in urban areas at levels once considered safe (Richter et al. 1980, 1986). Results of previous studies on childhood lead exposure in the Palestinian Authority, Jordan, and Israel are summarized in Table 1.

Investigation of an episode of familial lead poisoning led to the discovery that the source was flour contaminated by lead from metal fittings of the axle on the stone grinding wheel, not only in the index village but also in many others (Hershko et al. 1989). A similar episode later occurred in eight children of a family that owned a flour mill in Hebron (El Sharif et al. 2000).

We report the results of the childhood lead poisoning prevention study (1996–2000) in Israel, Jordan, and the Palestinian Authority supported by U.S. Agency for International Development (USAID)/Middle East Regional Cooperation–Centers for Disease Control and Prevention (West Bank and Gaza). The objectives of the study were *a*) to assess the distribution of PbB levels; *b*) to search for sources of exposure to lead among children in Israel, Jordan, Gaza, and the West Bank, including so-called “hot spots”; *c*) to assess the utility and practicality of screening, case finding, source identification, and prevention, using a new state-of-the art method for fingerstick (FS) measurement of PbB; *d*) to determine whether there is a need for routine surveillance and screening in each of the four regions; *e*) to determine whether reduction in use of leaded gasoline and total emissions resulted in reduction in PbB in children in the region; and *f* ) to promote appropriate interventions. The outbreak of political conflict and violence in September 2000 disrupted progress toward the last objective.

The four regions differ substantially from one another in terms of standard indices of development, socioeconomic status, and public health (Table 2). However, three of the four regions—Israel, West Bank, and Gaza—share a common source of fuel, and access to water in all four regions is closely interrelated. Lead emissions from stationary and mobile sources are also transported across boundaries as air pollution (Silbergeld 1995). Because socioeconomic status is often a predictor for lead exposure among children, we expected to find variations in PbB levels among the different regions. The advent of the LeadCare FS method offered new possibilities for cost-effective and efficient field investigations.

## Materials and Methods

### Population, study design, and sampling strategy

We chose children 2–6 years of age as the target population based on the assumption that their exposure to the outdoors and crawl zones would exceed that of younger infants. The project had two components. The first was a cross-sectional prevalence survey in all regions. The second component was measurement of PbB levels in children living near suspected hot spots—a situation that occurred in Gaza (see below). Each region was divided into subregions, which in turn were sampled. The testing was carried out in 1998–2000. A parent or guardian gave written and oral informed consent for each child, according the Institutional Review Board requirements.

For the prevalence study, the design and sampling methods took into account differing population distributions, age patterns, residential distribution, anticipated prevalence, patterns of clustering of high PbB levels, percentage of responders, and funding and time constraints. The investigators used a stratified two-stage cluster probability design for each of the regions. Each had different systems for defining and enumerating neighborhoods, houses, and children. In the Gaza and West Bank studies, the teams also identified areas near point sources of environmental lead contamination.

In Gaza, the project team divided the region into 48 primary sampling units (PSUs) and selected a fixed number of children from within each PSU. The main sources of exposure were smelters, battery recycling units, and other industries using lead. The team examined one child 2–6 years of age in the house located nearest the source or, if no source was found, nearest the main street. This task was repeated until the required number of children were sampled in each PSU.

In Israel, the team attempted to select areas identified as meeting at least one of the following criteria as a PSU: an area with heavy traffic density, a population from a lower socioeconomic standard, an area with old constructed houses, or an area with a point source of lead—for example, an industry using lead. In addition to PbB measurement, they measured zinc protoporphyrin (ZPP) in the study population. In Israel, major problems of access and response resulted in underrepresentation of urban areas, as well as possible underrepresentation of hot spots.

In the West Bank, the sources of lead pollution included industrial hot spots and traffic congestion. The PSUs were the 10 cities with populations ≥ 50,000. In each PSU in the West Bank, the team identified maternity and child health (MCH) centers administered by the government, United Nations Relief and Works Agency, or nongovernmental organization, located on or within 100 m of the heavy-traffic streets. If the team found a child to have PbB > 10 μg/dL, they took a repeat blood sample for confirmation.

In Jordan, the PSUs were nine governates, each with a population of > 100,000 inhabitants. In each of the governates, the team selected children from a proportional number of MCH centers located ≤ 200 m from the main streets.

To search for childhood exposures near suspect “hot spots” in each area, we set aside a number of examinations reserved for areas or situations designated as high-risk “hot spots”—for example, areas where we suspected increased exposures to lead. These also include children of adults employed in professions that are associated with exposure to lead.

### Information on sociodemographics, potential exposures, and health status

We developed a questionnaire, which was translated into Arabic, Hebrew, and Russian, for collection of information and pretested it in the field. Demographic information on the child; family status; household, dietary, and environmental sources of exposure; and health status was collected by one-on-one interviews based on the questionnaire. A follow-up study will report the distribution and determinants of PbB levels in relation to these sources.

### PbB and ZPP

All teams used the LeadCare kits (Esa Biosciences Inc., Chelmsford, MA, USA; Esa Biosciences Inc. 2005; Pineau et al. 2002), a validated method for accurately measuring lead levels in FS blood specimens. A 2-day training session was held for the field staff from all four regions on how to use the equipment in the field. The equipment remains available for use in each region after the completion of the project. For ZPP measurements, blood samples were collected from the same FS and analyzed using the Aviv Hematofluorometer (Aviv Biomedical Inc., Lakewood, NJ, USA). Blood samples were collected in schools, kindergartens, and nurseries.

### Quality assurance

We compared the results of the FS method versus venipuncture, based on specimens taken from consenting adults. Quality assurance/quality control assessments of the method determined the reliability, accuracy, and precision of the method in each region. The teams used the LeadCare Quality Control Sheet for testing standards (one low, one high) before and after each testing session.

### Response rate and selection biases

In all districts in the West Bank, Gaza, and Jordan, response rates were generally high among parents, but we could not calculate precise rates. In Gaza, it was necessary to modify the plan to include only one child from each household. In Israel, we had trouble gaining access to districts in urban areas with high traffic. In all areas, if parents asked that their other children be tested, we did the test but did not include results from these children in the reported results. We refused no child a blood test. The teams notified the families of the children found with high PbB levels and advised them to see their physician or hospital pediatric services.

### Time trends in lead emissions

Data on total lead emissions in Israel came from the Central Bureau of Statistics (Statistical Abstract of Israel 2004), and data on prior PbB levels in children came from previous studies, as listed in Table 1.

This project began in 1996; we completed the fieldwork in 1999–2000, the data analysis in 2001–2002, and writeups in 2004–2005.

## Results

The results of the pretest showed a high degree of reliability for the FS, but the β-coefficient for variance indicates a tendency toward slight systematic upward error in the West Bank and Gaza, with much higher systematic error in Israel, suggesting the possibility of skin surface contamination (Table 3). In Israel, the fact that the pattern of results was consistent with exposure circumstances led us to conclude that errors in accuracy and precision decreased during the project.

**Figure 1:**
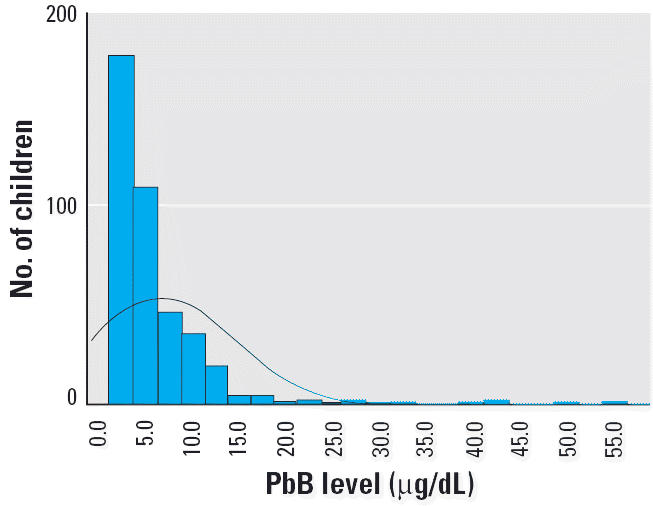
PbB levels in children in Gaza (excluding children with PbB > 60). Mean ± SD = 7.75 ± 6.8 μg/dL; *n* = 427.

**Figure 2:**
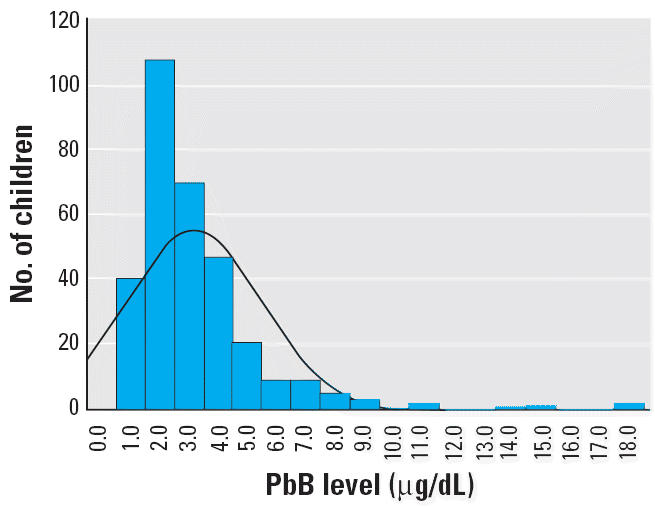
PbB levels in children in Israel. Mean ± SD = 3.2 ± 2.32 μg/dL; *n* = 318.

**Figure 3:**
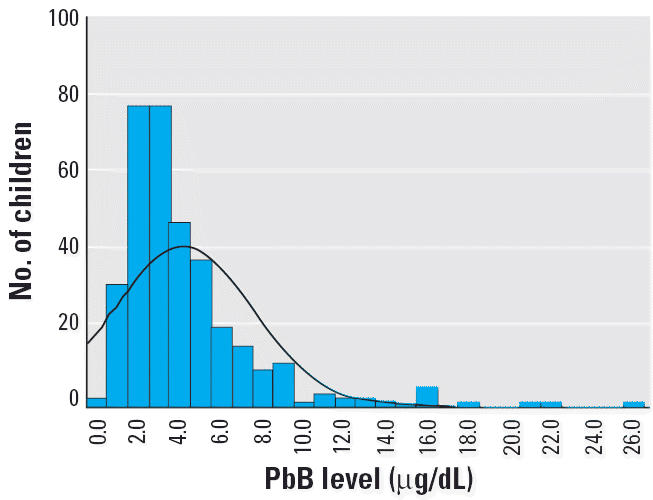
PbB levels in children in the West Bank. Mean ± SD = 4.2 ± 3.36 μg/dL; *n* = 344.

**Figure 4:**
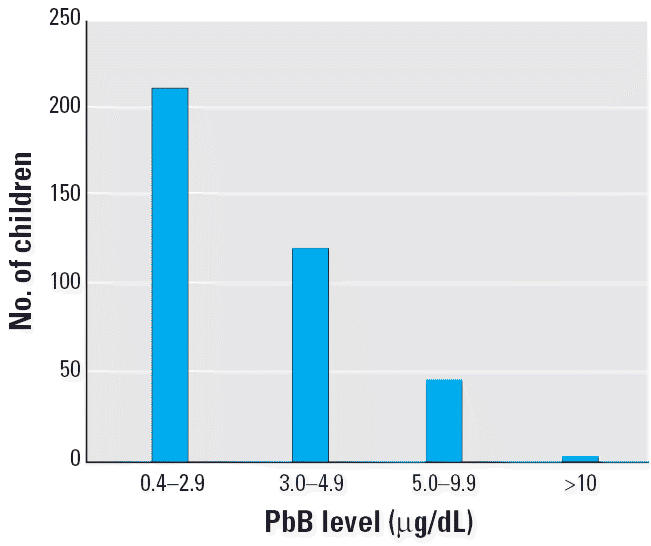
PbB levels in children in Jordan. Mean = 3.2 μg/dL; *n* = 382.

Table 4 and Figures 1–4 present the results of this study in each of the four regions. The findings show that the risk for PbB > 10 μg/dL in children 2–6 years of age was 2.2% in Israel, < 1% in Jordan, 5.2% in West Bank, and 17.2% in Gaza. In the West Bank and Gaza, the percentages of children with PbB levels between 5 and 9.9 μg/dL were 21.5 and 26.2%, respectively, compared with 14 and 12%, respectively, in Israel and Jordan. In Gaza, there were defined hot spots for risk related to proximity to local small-industry–type smelters engaged in secondary recycling, smelting, and battery manufacture. All the children in Gaza with PbB levels > 10 μg/dL lived in the vicinity of a battery factory or smelter. In the West Bank, although no extreme hot spots were detected, the relatively higher percentage of children with PbB levels > 5.0 μg/dL was noteworthy. Because only two of all those tested in the West Bank lived near a smelter or battery factory, the findings might suggest a higher representation of children living near high-traffic zones.

In Israel, 268 (84.5%) of 317 of children tested had PbB levels < 5 μg/dL (Table 4, Figure 2), with most of the values in the range of 2–2.9 μg/dL. Among children from Jewish areas, maximum values were highest in Jerusalem (4.7 μg/dL), Bnei Brak (8.9 μg/dL), and Haifa (8.0 μg/dL). There were higher mean, median, and maximum values in Bedouin and Druze areas, where children play more outdoors in less controlled settings, compared with Jewish areas. Children in Yarka, a Druze village far from urban air pollution, had the highest mean and maximum values. Seven children in Yarka had PbB > 0.0 μg/dL.

**Figure 5:**
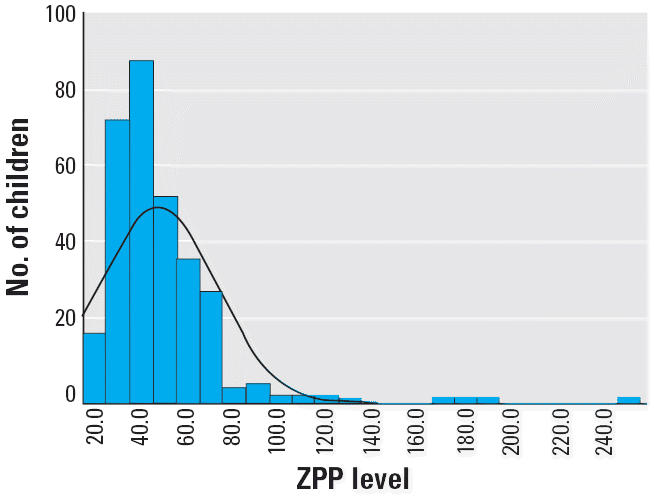
ZPP levels in children in Israel. Mean ± SD = 47.5 ± 25.10 (μmol/mol heme); *n* = 318.

Table 5 and Figure 5 present distributions for ZPP in Israel. Because PbB levels were generally well below the threshold for increased levels of ZPP, there was no correlation between elevation in ZPP and elevation in PbB (see “Discussion”).

**Figure 6:**
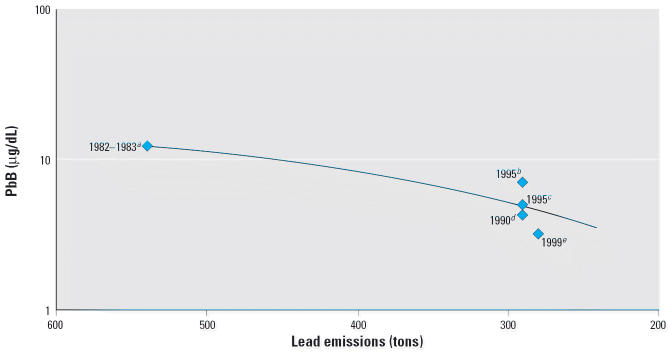
Lead emissions and PbB levels in children in Israel, 1982–1999. *R*^2^ = 0.8575. ***a****n* = 44 children in Haifa (mean = 12.4 μg/dL). ***b****n* = 206 children in East Jerusalem (mean = 7.2 μg/dL). ***c****n* = 94 children in Netanya (mean = 5.0 μg/dL). ***d****n* = 30 infants in Druze village, non-kohl users (mean = 4.3 μg/dL). ***e****n* = 317 in Israel (mean = 3.2 μg/dL). Data from Fischbeinet al. (1996); Nir et al. (1992); Richter et al. (1986); Statistical Abstract of Israel (2004).

Figure 6 shows the time trends in lead emissions from fuel combustion in Israel and PbB in children, based on prior studies and results from this study in Israel. Over a 17-year period, lead emissions had fallen 85% from 540 tons in 1985 to 90 tons in 2003. There is a high correlation (*r* = 0.85) between lead emissions and blood levels in children, in keeping with a relationship in which reduction of approximately 30 tons of lead emissions led to a drop of approximately 0.5 μg/dL, but the data show intercity differences in mean values in 1995 and 1999. Furthermore, within Israel, in Haifa and Jerusalem, two cities for which we had data from 1995, blood levels in children in our study were much lower (3.4 and 2.4 μg/dL, respectively) than those based on results from bloods collected in 1995 (Fischbein et al. 1996).

In Jordan, most (55%) of the children examined (*n* = 382) had PbB < 3 μg/dL, and three children (< 1%) had PbB > 10 μg/dL. In the West Bank, 18 (5.2%) of 344 children examined had PbB > 10 μg/dL (Table 4, Figure 3). Most children (73.3%) had PbB levels < 5 μg/dL, and 99.1% of the children did not live near a known point source of lead contamination.

Of the 435 children examined in Gaza, 75 (17.2%) had PbB ≥ 10 μg/dL, and 31 (7.1%) had PbB > 20 μg/dL. All 75 children with PbB > 10 μg/dL came from a subgroup of 108 (24.8%) children living adjacent to a smelter, a battery plant, or both, a finding that suggests some 60% of children living near these sites were at risk for lead poisoning. Table 6 shows the percentage of children in Gaza living near a source of lead pollution.

We analyzed the Gaza data on PbB in relation to living near sites of battery recycling, near a smelter, or near battery manufacturing (Tables 6, 7). There were strong associations between PbB levels and living near battery recycling [odds ratio (OR) = 13], a smelter (OR = 2.66), or battery manufacturing (OR = 9.5), and inverse correlations between blood levels and distance from these sites. In contrast, there was a positive correlation between PbB and distance from a major road (+ 0.204). These data strongly suggest the specificity of the association between PbB > 10 μg/dL and proximity to the point sources.

## Discussion

The present study, the largest multinational survey of its kind in the Middle East, achieved its major objectives: defining the current status of lead exposure in children in Israel, Jordan, and the Palestinian Authority; identifying hot spots; determining whether reduction in use of leaded gasoline resulted in reduction in PbB in children in the region; and introducing low-cost, reliable, and accurate state-of-the-art methods for determining PbB levels for purposes of surveillance, screening, targeted investigation and case finding.

Despite the major socioeconomic differences between Israel, Jordan, the West Bank, and Gaza, PbB levels in children 2–6 years of age were strikingly within the same range—except for children living near hot spots. The mean and geometric levels in the region are now approaching those of Northern Europe and the United States, where mean PbB levels in children range between 2.1 and 2.7 μg/dL (Pirkle 1998; Ponka 1998; Strömberg 2003; Wilhelm et al. 2002). Although this study was not a classic representative sample, the cluster design enabled us to sample groups throughout the country in many different exposure settings.

The findings, taken together with results of previous surveys in Israel, and a separate parallel study in Jordan (Dabbas and Al-Zoubi 2000) showing average levels of approximately 2.0 μg/dL, suggest that the decline in PbB levels in children in the region is attributable to the progressive phasing out of leaded gasoline. The 85% drop in lead emissions in Israel explains the fall of population-wide PbB levels in Israeli children from 14.3 μg/dL in the 1980s to 6.0 μg/dL in the mid-1990s, to those currently reported. Apart from hot spots, the mean PbB levels were approximately comparable in all regions. It therefore is plausible that the association between decreased lead emissions and decreased PbB levels in children in Israel parallels trends in the entire region.

We discovered a major hot spot in Gaza. We cannot rule out the possibility of other hot spots, especially in poorer areas, or high-risk situations, for example, among children of radiator repair workers, as occurred in Israel.

### Lead and ZPP

The lack of correlation between PbB and ZPP in Israel was expected because the PbB level was below the threshold for inducing elevation of ZPP. Therefore, it is reasonable to suggest that where ZPP levels were increased, iron deficiency may be the cause. Because 0–2 years is the peak age group for iron deficiency, we were not reaching the high-risk group by age. Our findings suggest the need to investigate the hypothesis that a much larger percentage of children in the 1-to 2-year age group are at risk for iron deficiency, not only in Israel but also in the West Bank, Gaza, and Jordan.

### Limitations

The project continued throughout the political upheavals and episodic violence in the region. However, follow-through on all the objectives, notably, promoting intervention targeted at the point sources, was disrupted.

There were several limitations of this study. In Israel, inner cities were underrepresented. Health officials were reluctant to mobilize support for a community survey based on blood tests for which there was a low probability of detection of clinical illness in individual children. The overall results were driven by the overrepresentation of one village. Also, response and participation of children from families living along roads with heavy traffic were low. We had problems gaining access to at least one major hot spot in Israel—600 children in a school near a smelter battery factory in the north of the country (Kfar Vradim), who, at the time of this writing, reportedly had been exposed to lead in previous years. The risks from these exposures have never been studied.

In Gaza, there were problems with follow-through in investigating and promoting interventions to abate the exposures from the lead smelter and battery plant.

Except for Gaza, the sampling strategies aimed at defining a so-called representative study may have led to skipping over hot spots or higher-risk areas and therefore may have produced underestimates of risks from focal point sources. Even so, the modified cluster design enabled us to look at the mean, median, geometric mean, and range of PbB levels in an array of settings.

Another limitation, leading to possible underestimates, is that we could not gain access to children < 2 years of age, the age at which PbB peaks in the United States. This is an age group in which hand-to-mouth routes disproportionately contribute to PbB burdens. On the other hand, older children have more exposure outside the household, and therefore, their levels better reflect airborne lead levels. A subsequent study will report lead levels in age subgroups (Witt J, unpublished observations).

In all regions, we were unable to directly carry out follow-up by confirming PbB > 10 μg/dL with a venous sample.

Finally, the study did not examine interactions between exposures to lead and iron deficiency, a problem that may be widespread among the youngest children.

### Reduction of exposure

This study documents the results of substantial progress in reducing lead in gasoline in the region, based on comparison with results of PbB in earlier years. However, in both Israel and Jordan, leaded gasoline has not been completely phased out. We recommend expediting the transition to lead-free gasoline, but note with great concern the new-generation substitute additives (e.g., methyl *tert*-butyl ether, platinum, manganese) with known toxic risks, whose public health effects have not yet been defined and that remain point source occupational exposures to fuel attendants (Ahmed 2001; Normandin et al. 2002).

### Surveillance and case finding

Findings suggesting the absence of a detectable threshold for neurobehavioral effects in children indicate the possibility of population-wide risks for children from exposures < 5 μg/dL. But as leaded gasoline continues to be phased out, it is not certain that there is a case for routine mass screening of all children in Israel, Jordan, and the West Bank. But there is a case for surveillance and episodic spot checks, as part of national surveys and screening within the framework of a comprehensive approach to child health and development, preferably as part of the currently operating national nutrition and child health surveillance system. In Gaza, where we estimate that there were at least several thousand children residing in neighborhoods close to hot spots and industrial point sources for lead, there is a case for targeted epidemiologic investigation of risks and intervention.

Overall, we suggest that sampling strategies should be focused on high-risk areas and so-called hot spots. Sampling strategies aimed at obtaining a so-called true representative level may divert scarce resources from identifying these high-risk areas or hot spots. We recommend that the priority target areas should be hot spots near smelters, battery recycling, and battery repair; corridors along heavy trafficked roads; and children of workers with occupational exposures to lead.

Targeted surveillance for lead, along with iron deficiency, should be part of primary health care for children. Today, examinations can be based on collection from one FS of several drops of blood for hemoglobin, ZPP, and PbB. In all regions, there is a case for equipping several primary medical care centers with the LeadCare kit and ZPP meter for surveys, selective screening targeted at suspect or special situations, and investigations triggered by sentinel cases. There remain problems with activating follow-through investigations for sources in all the four study areas.

### Industrial hygiene

There is a need for a uniform transborder regional level of lead protection based on best possible industrial standards. To achieve this goal, there is a need for a major investment in industrial upgrading, training, and surveillance. An earlier study demonstrated high levels of lead poisoning and neurotoxic damage in lead battery workers from Gaza working in an Israeli smelter (Hashish M and Richter ED, unpublished data).

In all regions, there is a need to gain access to children at risk of exposure from industrial sources, either by proximity to point sources or as children of workers. There is a need to activate labor inspectorates to trace worker populations at risk. Projects to identify children at risk should be used to locate workers at risk.

In conclusion, we recommend the following actions: *a*) surveillance and episodic checks at “hot spots,” including corridors along heavily trafficked roads and battery factories, smelters, and auto radiator repair; *b*) targeted surveillance for lead, iron deficiency, and ZPP in all regions; *c*) equipping primary medical care centers in each region with LeadCare kits and ZPP meters; and *d*) reducing lead emissions from industrial sources of lead pollution, especially in Gaza.

## Correction

The order of authors has been changed from that in the original published online, and two co-authors, Matti Bercovitch and Efim Platkov, have been added.

## Figures and Tables

**Table 1:** Previous studies on lead exposure in the Palestinian Authority, Jordan, and Israel.

Year	Population	PbB (μg/dL)	Reference
1982	Children, Haifa Bay	ZPP < 40 μg/dL PbB mean, 12.1 ZPP > 40 μg/dL PbB mean, 15.5	Richter et al. 1986
1983	Teachers, Jerusalem	8.6 (median)	Friberg and Vahter 1983
1990	Children and infants, Druze village	Mean, 4.3; mean kohl users, 11–12	Nir et al. 1992
1995	Children, Netanya Hospital	Mean, 6.0; 9% > 10 μg/dL	Fischbein et al. 1996
1995	Children, East Jerusalem	Mean, 7.0; 80% > 5 μg/dL	Fischbein et al. 1996
2000	Jordanian population (representative sample)	Mean, 1.96	Dabbas and Al-Zoubi 2000

**Table 2:** Statistical information for Jordan, West Bank and Gaza, and Israel.

	Jordan	West Bank and Gaza	Israel
Population (millions)	4.91	3.12	6.33
Percent population < 5 years of age	15.04	18.52	9.15
Illiteracy (% > 15 years of age)	4.9 M	10.23	3.2 M
	16.1 F		7.6 F
GDP per capita (US$)	1693.91	1484.52	17523.81
Infant mortality (no. per live 1,000 births)	25.31	22.01	5.51
Population with access to improved water sources (%)	96.0	84.8	99.0
Population with piped sewage (%)	100	34 WB	NA
		53.5 G	
Unemployment (%)	NA	47	7.7 (1997)
Poverty (%)	12	55 (2000)	18 (1999)
Urban population (%)	74.2	NA	91.2
Water consumption (L/capita/day)	NA	706	3,506

Abbreviations: F, female; G, Gaza; GDP, gross domestic product; M, male; NA, not available; WB, West Bank. Data are relevant for the study period 1999–2000 (Jordan Department of Statistics 1994; Lein 2000; National Insurance Institute 2000; Palestine Central Bureau of Statistics 2000; Palestinian National Authority Ministry of Health 2001; Statistical Abstract of Israel 2001; World Bank 2000).

**Table 3:** Results of the pretest in consenting adults: FS method (*y*) compared with venous stick (*x*) and β-variance.

	Israel	West Bank	Gaza
*Y*	0.265*x* + 3.227	0.763*x* + 1.083	0.5714*x* + 0.662
*R*	0.42	0.89	0.84

**Table 4:** Summary findings: Gaza, Israel, West Bank, and Jordan, 1999–2000.

	Gaza	Israel	West Bank	Jordan
No.	435	317	344	382
Geometric mean (μg/dL)	5.2	2.7	3.3	1.4
Mean (μg/dL)	8.6	3.21	4.20	3.22
Median (μg/dL)	4.3	2.6	3.3	2.9
Range (μg/dL)	0.5–124.4	0.4–18.2	0.4–25.7	0.4–39.3
No. (%), 5–9.9 μg/dL	114 (26.2)	43 (14.0)	74 (21.5)	46 (12.0)
No. (%), ≥ 10 μg/dL	75 (17.2)^a^	7 (2.2)	18 (5.2)	3 (0.8)

PbB values > 60 μg/dL were estimated using a dilution technique.

a All among children living in the vicinity of a battery factory or smelter.

**Table 5:** PbB and ZPP levels in 317 Israeli children.

	PbB (μg/dL)					ZPP (μmol/mol heme)			
Location	No.	Mean	Median	Minimum	Maximum	Mean	Median	Minimum	Maximum
Urban									
Beer Sheva	7	1.93	1.7	1.4	2.7	62.86	65	30	112
Bnei Brak	57	2.6	2.3	1.3	8.9	56.93	49	6.10	250
Haifa	11	3.36	2.9	1.2	8.0	46.91	48	29	75
Herzliya	5	2.14	2.2	1.6	2.9	44.60	47	27	70
Jerusalem	11	2.39	1.7	0.9	4.7	55.36	48	1.0	80
Petach Tikveh	1	NA	NA	2.0	2.0	NA	NA	72	72
Rural near smelter									
Kisra (Druze)	19	3.32	3.5	1.3	7.5	32.37	27	18	125
Kfar Vradim	22	2.50	2.4	1.0	6.5	29.90	28	20	49
Rural									
Maalot	2	1.9	1.9	1.8	2.0	32	32	28	36
Nof Yam	6	2.2	1.95	1.4	3.5	37.83	35	25	64
Araar (Bedouin)	18	2.64	2.05	1.3	6.5	50.67	43.5	28	110
Yarka (Druze)	157	3.79	3.1	0.4	18.2	47.56	42	23	180
Mitzpe Ramon	1	NA	NA	0.80	0.80	NA	NA	25	25

NA, not available.

**Table 6:** PbB levels in children in Gaza, according to proximity to lead contamination sources: smelter, battery manufacturing plant, and battery recycling plant.

Proximity to contamination source	No. of children (%)	PbB (μg/dL) [mean (range)]	PbB > 10 μg/dL [no. (%)]	OR
No contamination sources	324 (74.5)	4.9 (4.5–5.3)	24 (7.4)	1.0
One contamination source	33 (7.6)	8.7 (4.7–12.7)	6 (21.7)	3.3
Two contamination sources	61 (14.0)	14.9 (11.1–18.7)	30 (50.8)	12.9
Three contamination sources	14 (3.2)	13.5 (6.8–20.2)	4 (33)	6.3
Missing	3 (0.7)	—	—	—

**Table 7:** Risk from living near source of lead exposure in Gaza: correlation coefficient.

Study factor	No. of children PbB < 10 μg/dL	PbB > 10 μg/dL	OR	Distance from source (*r*)
House near major road				+0.204
House near battery recycling				
No	312	25	13 (*p* < 0.00)	−0.512
Yes	40	50		
House near smelter				
No	346	65	2.66 (*p* = 0.03)	−0.392
Yes	14	7		
House near battery manufacturing				
No	323	36	9.5 (*p* < 0.000)	−0.501
Yes	37	39		
